# Heavy load chain squats: a promising method for enhancing lower extremity explosive strength via post-activation performance enhancement

**DOI:** 10.3389/fphys.2025.1596200

**Published:** 2025-06-18

**Authors:** Dianxue Wu, Shuo Feng, Aiguo Zhou, Yongmin Xie

**Affiliations:** School of Strength and Conditioning Training, Beijing Sport University, Beijing, China

**Keywords:** Post-Activation Performance Enhancement, Chain Squats, Countermovement Jump, Explosive Strength, Resistance Exercise

## Abstract

**Introduction:**

Post-Activation Performance Enhancement (PAPE) is widely used to enhance explosive performance. Chain Squats (CS), as a form of variable resistance training, may offer advantages over Traditional Squats (TS) due to its load variability and neuromuscular stimulation characteristics. However, direct comparisons between the two under controlled conditions remain limited. Therefore, this study aims to compare the effects of CS and TS on PAPE to explore their different impacts on explosive strength.

**Methods:**

Eighteen male participants completed CS and TS training at 90% of their 1RM in a randomized crossover design. Countermovement Jump (CMJ) tests were conducted at baseline and at 4, 8, 12, and 16 min post-intervention to evaluate Jump Height (JH), Peak Power (PP), Peak Rate of Force Development (PRFD), and Peak Impulse (PI).

**Results:**

Both CS and TS significantly enhanced explosive performance, but CS outperformed TS in several metrics. CS showed greater improvements in JH at 4 min (P < 0.01, η^2^ = 0.119, d = 3.69) and 8 min (P < 0.01, η^2^ = 0.102, d = 3.49). For PP, CS also outperformed TS with larger effect sizes at the same time points. PRFD showed no significant change in either modality, but CS showed a downward trend at 12 min (P = 0.055, η^2^ = −0.014, d = −424.16). PI peaked at 4 min in the CS group (P < 0.01, η^2^ = 0.019, d = 6.56), significantly outperforming TS.

**Discussion:**

CS significantly outperformed TS in enhancing JH, PP, and PI during the 4-8 min post-intervention period. Compared to TS, CS is a more advantageous PAPE strategy, offering a more effective way to enhance explosive performance in athletes.

## Introduction

Enhancing athletic performance through scientifically grounded and time-efficient strategies remains a central issue in contemporary training practice and sports science research. In recent years, PAPE has gained considerable attention as a method capable of acutely improving voluntary strength, explosive power, and speed within a matter of minutes (Blazevich and Babault, 2019; Tillin and Bishop, 2009).

PAPE is typically defined as a transient enhancement in voluntary performance following a bout of moderate-to high-intensity conditioning contraction (CC) (Cuenca-Fernández et al., 2017). In practical applications, PAPE strategies have been widely utilized in the design of warm-up routines, pre-competition activation, training volume optimization, and explosive power development (Seitz and Haff, 2016; Maroto-Izquierdo et al., 2020). According to Boullosa’s summary of relevant studies (Boullosa, 2021), effective induction of PAPE generally requires resistance exercises performed at an intensity ≥70% of one-repetition maximum (1RM), with 2–3 sets and a moderate number of repetitions, in order to sufficiently activate the neuromuscular system while minimizing excessive fatigue.

Among various resistance exercises, the Traditional Squat (TS)is widely recognized as a classical and effective modality for eliciting PAPE responses, due to its adjustable loading parameters and the involvement of multiple joints (Vargas-Molina et al., 2021; Heynen et al., 2024; Wilson et al., 2013). It has been demonstrated to significantly enhance subsequent performance and is therefore broadly applied across a wide range of athletic disciplines (Lowery et al., 2012). However, during traditional squat exercises, the external resistance remains relatively constant throughout the movement, while the muscle’s force-generating capacity varies with changes in joint angles. According to the force–velocity relationship (Wallace et al., 2018; Alcazar et al., 2019), peak force during traditional squats typically occurs near the terminal (top) phase of the movement, where joint angles are more mechanically advantageous and barbell velocity increases. At this stage, the muscles operate under an optimal length–tension relationship and benefit from more favorable leverage conditions, allowing for near-maximal force output even at higher contraction velocities. This phenomenon illustrates the practical implication of the force–velocity relationship during the top phase of a traditional squat: the joints are positioned in a biomechanically efficient configuration, enabling substantial force production despite the increased movement speed (Tkuasl, 2021). Studies have shown that (Friedman, 2016), during traditional squats exercises, a region commonly referred to as the “sticking point” often occurs in the ascending phase of the movement. This region typically appears in the mid-range of the lift, where joint angles are biomechanically disadvantageous, making it difficult for muscles to produce effective force. As a result, trainees are more likely to experience movement failure or technical breakdown at this stage. The associated reduction in force output and deceleration of movement compromise the overall efficiency of the lift, thereby limiting training quality and hindering performance improvement (Kompf and Arandjelović, 2016).

At this point, the body may struggle to overcome external resistance, limiting the effectiveness of the stimulus and potentially failing to optimally induce PAPE (Friedman, 2016; Esformes and Bampouras, 2013; McMaster et al., 2009). In response to this issue, Louie Simmons proposed Variable Resistance Training (VRT), which combines fixed weights with chains (Simmons, 2025). Chain squats (CS) involve using a barbell with chains attached to both ends. As the barbell descends, the chains’ weight on the ground reduces the load; as the barbell ascends, the load increases as the chains lift off the ground (Berning et al., 2008). This chain-loaded squat method may address the limitations of traditional barbell training by providing a progressively increasing load that better matches cumulative muscle force and rising joint torque throughout the concentric phase. Compared to TS, CS may extend the period of muscle activation, providing optimal loading across a broader range of motion, thus better reflecting the muscle length-tension relationship during the exercise (Wallace et al., 2018; McMaster et al., 2009; Wallace et al., 2006; Findley, 2004). Additionally, chain training has shown positive effects on long-term gains in both explosive power and maximal strength. In a long-term intervention study, Salvador et al. demonstrated that CS improved explosive power and increased the rate of force development more than traditional training (Baena Morales et al., 2022). Minas et al. used 85% 1RM CS as a warm-up method and found that CS produced a greater 1RM increase compared to TS (Mina et al., 2016). Although CS have demonstrated advantages in both long-term interventions and acute studies, and are theoretically more conducive to eliciting PAPE for improving athletic performance, empirical research on their application remains limited. Therefore, the present study aims to compare the PAPE-inducing effects of CS and TS at multiple post-intervention time points and to assess the feasibility of CS as a form of CC using kinematic indicators. By identifying the optimal timing for intervention, this study seeks to provide scientific evidence for optimizing warm-up strategies and developing individualized training protocols, expanding warm-up and daily training methods for athletes in explosive power events.

Based on previous studies on banded squats and CS, as well as preliminary experimental data, a load of 90% 1RM with a 20% chain weight ratio was selected for CS(17, 20). It is hypothesized that the CS condition would induce a greater PAPE response and lead to superior improvements in explosive performance compared to the TS condition, particularly at specific time points following the conditioning activity.

## Materials and methods

### Experimental approach

This study employed a randomized crossover experimental design to evaluate the effects of different CS loads on PAPE. Each participant first underwent a 1RM deep squat test, followed by two distinct experimental intervention sessions. The primary objective was to examine how varying CS loads influenced PAPE and to compare these effects with those of TS.

### Subjects

The sample size was calculated using G*Power 3.1, assuming a medium effect size for the primary outcomes. The following parameters were set: a significance level (α) of 0.05, a statistical power (1−β) of 0.80, and a repeated-measures design with an estimated intermeasure correlation coefficient of 0.7. Based on these calculations, the required sample size was determined to be 16 participants. To account for potential data attrition and participant dropout, a final total of 18 male collegiate sprinters from Sport University were selected to participate in the study. All participants were certified sprint athletes, each holding a national second-class or higher athletic qualification as recognized by the General Administration of Sport, and had achieved top-three rankings in individual or relay sprint events at provincial-level or higher competitions, basic information is shown in Table 1.

**TABLE 1:** Basic information on the participants ( $\bar{x}$ ±s).

Height (cm)	Body Weight (kg)	Ages (years)	Years of training (years)	Deep Squat 1RM (kg)
182.19 ± 5.15	76.05 ± 7.37	21.77 ± 1.66	4.27 ± 0.89	139.72 ± 16.31

RM, repetition maximum.

All participants had at least 2 years of resistance training experience, demonstrated proficiency in standardized deep squats, and regularly trained with loads exceeding 1.5 times their body weight. To ensure they were physically prepared for the experimental procedures, participants were free from chronic health conditions, psychiatric disorders, and lower extremity injuries.

Informed consent was obtained from all participants after a thorough explanation of the experimental protocol. Pre-exercise health screenings were conducted to minimize potential health risks. This study was approved by the Ethics Committee (2025083H) and adhered to the guidelines of the Declaration of Helsinki.

### Chain squat load calculation

**FIGURE 1:**
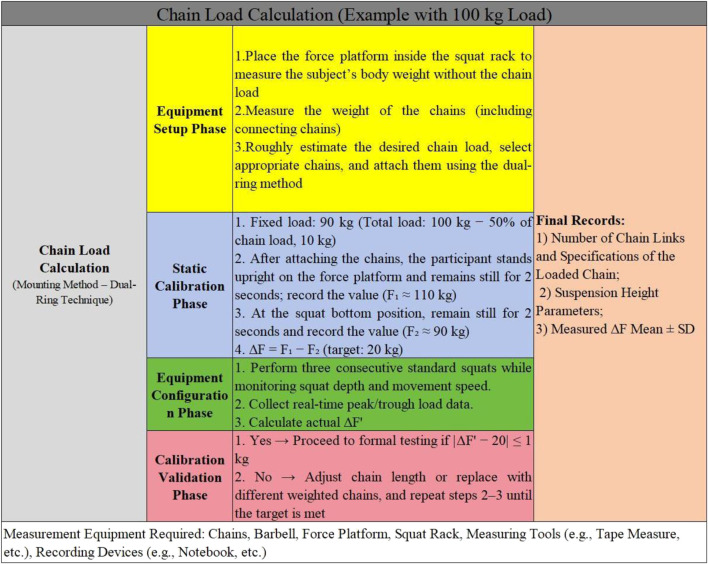
Chain load measurement procedure.

For CS, using an intervention weight of 100 kg as an example, the chain weight should be 20 kg. This means the load variation from the squat’s lowest position to full standing should be 20 kg. To ensure that the total load during the CS matched the 100 kg load used in the TS, the fixed load (i.e., the barbell and weight plates) was adjusted accordingly. Specifically, the fixed load was calculated as the target load minus 50% of the chain weight, resulting in a fixed load of 90 kg (i.e., 100 kg − 20 kg × 0.5). A force platform was positioned within a power rack, and chains were attached to both ends of the barbell using the dual-ring method. To minimize chain sway during squats, part of the chain remained in contact with the ground when the participant was in the upright position. Participants stood on the force platform in a standard squat stance, lifted the barbell, and held it steady for 2 s to record the weight at this point. They then performed a squat to the lowest position, paused for 2 s, and recorded the weight again. This process was repeated at least three times to ensure accuracy, with the average taken from the highest and lowest values. The difference between these averages was expected to approximate 20 kg. If the discrepancy was too large or small, the chain weight and length were adjusted until the difference was close to 20 kg. Finally, the specifications, number, and placement of the chains were recorded (McMaster et al., 2009; Baker and Newton, 2009; Liming et al., 2022),the specific procedure for chain load measurement is shown in Figure 1.

### Procedures

#### 1RM squat test

Participants completed a deep squat 1RM test 5 days prior to the formal intervention, following a standardized protocol from the National Strength and Conditioning Association (Friedman, 2016). The testing procedure began with a warm-up, during which participants performed 5–10 repetitions with a moderate load, followed by a 1-min rest. For the first load increment, 10%–20% of the initial weight was added, and participants completed 3–5 repetitions, followed by a 2-min rest. A second load increment of 10%–20% was then applied, with participants performing 2–3 repetitions and resting for 2–4 min thereafter. During the formal 1RM attempts, the load was increased by 10%–20% for each trial, with 2–4 min of rest between attempts. If a participant failed to successfully complete a 1RM attempt, the load was reduced by 5%–10%, and the attempt was repeated after a 2–4 min rest interval. The 1RM determination was typically completed within five attempts, with successful lifts defined by proper squat technique.

During testing, participants were required to descend until the thighs were parallel to the ground, then ascend while maintaining a stable trunk posture to ensure the validity of the results. To improve measurement accuracy, each participant completed two separate 1RM squat tests (with an inter-test interval >72 h), and the highest value was used to determine the training load for subsequent interventions. This standardized testing protocol ensured accurate 1RM assessment for all participants, providing a precise reference for load prescription in the intervention phase.

#### PAPE-inducing intervention

Previous studies have shown that performing heavy weight-bearing deep squats is more likely to elicit a stronger PAPE effect (Lowery et al., 2012; Fukutani et al., 2014). Pre-experimental trials in this study indicated that chain sway could be minimized under heavy load conditions, which is why 90% of the 1RM was chosen as the intervention load for both TS and CS. In TS, the load was provided by a barbell and weight plates, while in CS, 80% of the total load was fixed, with the remaining 20% provided by chains.

Before each workout, participants began with a standardized 8–10 min warm-up, which included jogging, muscle activation, dynamic stretching, and movement integration. After completing the warm-up, participants rested for 3 min before the pre-test. They then performed four sets of progressively loaded warm-up squats: 6-8 repetitions using an empty bar, followed by five reps at 30% 1RM, 3 reps at 50% 1RM, and 2 reps at 65% 1RM, with 2-min rest intervals between each set. After an additional 3-min rest, the formal intervention began. During the intervention, participants were required to complete 3 sets of 2 repetitions for each exercise, regardless of whether they were performing TS or CS, for a total of 6 squat repetitions. A 3-min rest was taken between each set. During the squats, participants were instructed to descend until their thighs were parallel to the ground, with experimenters supervising to ensure proper form. To ensure participant safety, at least two experimenters were present to provide support during each exercise, in case of emergencies due to fatigue.

PAPE is highly time-dependent and typically exhibits an inverted U-shaped temporal response. Although numerous studies have reported that recovery intervals exceeding 5 min are generally more conducive to eliciting PAPE effects (Boullosa, 2021), the optimal recovery window is closely related to the type of performance task. For instance, peak improvements in jump performance have been observed between 4.5 and 6.3 min, whereas sprint performance may peak later, between 3.6 and 8.6 min (Xu et al., 2025). Other studies have also shown that enhancements in performance can occur within recovery periods shorter than 5 min (Chaouachi et al., 2011; Maloney et al., 2014).

However, high-intensity CA often induce considerable acute fatigue, particularly within the early recovery phase, which may mask potential performance enhancements, thereby affecting the observable PAPE response. Previous research has indicated that, especially in well-trained individuals with higher strength levels, a 4-min recovery interval may effectively mitigate fatigue while maintaining a relatively high level of neuromuscular excitability (Blazevich and Babault, 2019). Furthermore, several studies employing chain-loaded variable resistance training as a CA stimulus (Mina et al., 2016; Mina et al., 2019) have reported significant improvements in jump performance within 3–4 min post-intervention. These findings further support the choice of the 4-min interval in the present study.

**FIGURE 2:**
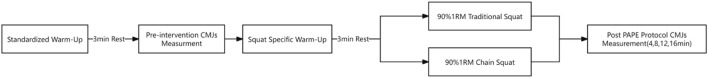
PAPE Testing Flowchart. PAPE, post-activation performance enhancement; RM, repetition maximum; CMJ, countermovement jump.

Therefore, a 4-min recovery interval was selected as the first post-CA time point in this study to balance the interplay between fatigue and potentiation, enabling more precise detection of the PAPE response. Given that countermovement jump (CMJ) performance was used as the primary outcome measure in this study, and that the participants had substantial experience in resistance training, a 4-min recovery interval was selected. CMJ tests were conducted at 4, 8, 12, and 16 min post-intervention. This timing was designed to allow for partial fatigue dissipation while maintaining an elevated level of muscle excitation, thereby optimizing the conditions for observing the PAPE effect and enhancing both the sensitivity of detection and the reliability of the results. The detailed testing protocol is illustrated in Figure 2.

#### CMJ Test

The CMJ test is a commonly used method to assess lower limb explosive strength. In this study, the portable dual-force platform (VALD FDlite, VALD Performance, Australia) was utilized to conduct CMJ tests before the intervention and at 4, 8, 12, and 16 min post-intervention. During testing, participants stood upright on a dual-force platform with their feet shoulder-width apart, hands placed on their hips, and torso maintained in an erect position. After baseline data were recorded, participants performed a rapid downward countermovement by flexing the knees to approximately 90°, followed immediately by a maximal vertical jump without pause. Each participant completed three valid trials. Arm swing was strictly prohibited throughout the test to ensure standardization and measurement reliability.

Based on previous studies and pre-experimental findings, the primary outcome measures selected for this study included jump height (JH, calculated based on impulse), peak power (PP), peak impulse (PI), and peak rate of force development (PRFD). A three-dimensional force platform was utilized to collect comprehensive kinematic and kinetic data for subsequent analysis.

#### Statistical analyses

The data collected in this study were analyzed using SPSS 26.0 software. A two-factor repeated measures analysis of variance (ANOVA) was conducted to examine the effects of the interventions (CS vs TS) and test times (pre- and post-intervention). A simple effects analysis was performed to compare differences in the measured indicators at each time point before and after the intervention. All statistical results are presented as mean ± standard deviation (±SD), with a significance level (α) set at 0.05. Results with a P-value less than 0.05 were considered statistically significant. To further assess the magnitude of the effects observed in this study, the effect size index η^2^ was calculated using RStudio software. This allowed for a quantitative evaluation of the impact of different loads in both CS and TS on explosive strength. Based on established guidelines for interpreting effect size (Shen et al., 2019), η^2^ values were categorized as follows: 0.010 to 0.059 indicated a small effect, 0.059 to 0.138 indicated a medium effect, and values greater than 0.138 were considered indicative of a large effect.

## Results

### Height

The Shapiro-Wilk normality test confirmed that the data in this group were normally distributed. Therefore, a two-factor repeated measures ANOVA was conducted. Mauchly’s test for sphericity yielded a P-value of less than 0.01, indicating a violation of the sphericity assumption. Therefore, the multivariate test results were prioritized, as they are considered more reliable. The analysis revealed a significant effect of different interval times on JH (F = 25.19 (5,64), *P* < 0.05, η^2^ = 0.62). No significant interaction was observed between the interventions and the interval times (F = 1.129 (15,198), *P* > 0.05, η^2^ = 0.079). Additionally, no significant differences in JH were found between the two interventions (F = 0.468, P > 0.05, η^2^ = 0.020).

**FIGURE 3:**
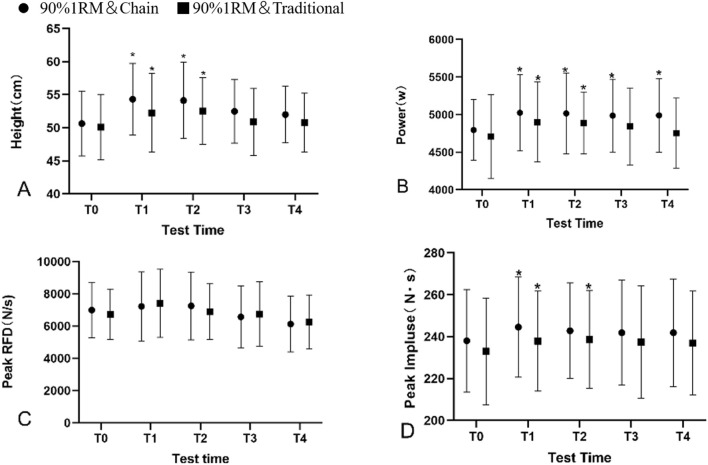
Separate comparative analyses of the two interventions (90% 1RM chain squat; 90% 1RM conventional squat) were performed at four post-intervention time points: 4 min (T1), 8 min (T2), 12 min (T3), 16 min (T4) for free height **(A)**, peak power **(B)**, peak RFD **(C)** and peak impulse **(D)** were different from the pre-test (TO) values. * Indicates that the value measured at that time point is statistically different from the baseline value. RM, repetition maximum.

Further pairwise comparisons using Sidak’s method (see Tables 2, 3; Figure 3A) revealed significant differences in JH at the 4-min and 8-min intervals following the 90% 1RM CS and TS interventions, compared to pre-test values (*P* < 0.05). For the 90% CS intervention at 4 min, η^2^ = 0.119, 95% CI [0.003, 0.301]; difference = 3.69 cm; at 8 min, η^2^ = 0.102, 95% CI [0.000, 0.280]; difference = 3.49 cm. For the 90% TS intervention at 4 min, η^2^ = 0.040, 95% CI [0.000, 0.192]; difference = 2.17 cm; at 8 min, η^2^ = 0.059, 95% CI [0.000, 0.223]; difference = 2.44 cm. Effect size indicators and difference comparisons (see Tables 2, 3) demonstrated that the 4-min interval following the 90% 1RM CS intervention had the most pronounced impact on JH, with the greatest increase observed (η^2^ = 0.119, 95% CI [0.003, 0.301]; difference = 3.69 cm).

**TABLE 2:** Post-intervention effects of chain squatting on indicators of η^2^.

Test Indicators	Intervention load	4 min VS Pre-test	8 min VS Pre-test	12 min VS Pre-test	16 min VS Pre-test
JH	90%1RM & Chain	0.119	0.102	0.036	0.022
	90%1RM & Traditional	0.040	0.059	0.006	0.005
PP	90%1RM & Chain	0.063	0.054	0.047	0.047
	90%1RM & Traditional	0.032	0.034	0.016	0.002
PRFD	90%1RM & Chain	0.004	0.005	0.014↓	0.063↓
	90%1RM & Traditional	0.034	0.003	0.000	0.022↓
PI	90%1RM & Chain	0.019	0.011	0.007	0.006
	90%1RM & Traditional	0.010	0.014	0.008	0.006

↓ indicates that the time point is in negative growth compared with the baseline value. JH, jump height; PP, peak power; PRFD, peak rate of force development; PI, peak impulse.

**TABLE 3:** Differences from pre-test at various interval times.

Test Indicators	Intervention load	4 min VS Pre-test	8 min VS Pre-test	12 min VS Pre-test	16 min VS Pre-test
JH	90%1RM & Chain	3.69	3.49	1.83	1.33
	90%1RM & Traditional	2.17	2.44	0.79	0.67
PP	90%1RM & Chain	230.49	220.89	191.56	194.78
	90%1RM & Traditional	179.78	192.67	134.00	43.83
PRFD	90%1RM & Chain	224.33	225.00	−424.16	−870.83
	90%1RM & Traditional	679.94	174.39	17.00	−475.33
PI	90%1RM & Chain	6.56	4.89	3.91	3.84
	90%1RM & Traditional	4.82	5.69	4.44	3.94

JH, jump height; PP, peak power; PRFD, peak rate of force development; PI, peak impulse.

### Peak power

The Shapiro-Wilk test confirmed the normality of the data, allowing for further analysis using a two-factor repeated measures ANOVA. Mauchly’s test indicated a violation of the sphericity assumption (*P* < 0.01), so the multivariate test results were considered more reliable. The analysis revealed a significant effect of interval times on CMJ PP (F = 17.29 (5,64), *P* < 0.05, η^2^ = 0.573), but no significant interaction was found between the interventions and interval times (F = 0.887 (15,198), *P* > 0.05, η^2^ = 0.063). Additionally, no statistically significant differences in CMJ PRFD were observed between the two interventions (F = 0.259, *P* > 0.05, η^2^ = 0.011).

Pairwise comparisons using the Sidak method (see Tables 2, 3; Figure 3B) showed that PP at the 4, 8, 12, and 16-min intervals following the 90% 1RM chained deep squat intervention was significantly higher than pre-test values (*P* < 0.05). For the 90% CS intervention, at 4 min, η^2^ = 0.063, 95% CI [0.000, 0.228]; difference = 230.49 W; at 8 min, η^2^ = 0.054, 95% CI [0.000, 0.215]; difference = 220.89 W; at 12 min, η^2^ = 0.047, 95% CI [0.000, 0.203]; difference = 191.56 W; at 16 min, η^2^ = 0.047, 95% CI [0.000, 0.202]; difference = 194.78 W. A similar pattern was observed following the 90% 1RM traditional squat (TS) intervention, where PP at the 4-min and 8-min intervals was significantly higher than pre-test values (*P* < 0.05). For the 90% TS intervention, at 4 min, η^2^ = 0.032, 95% CI [0.000, 0.178]; difference = 192.67 W; at 8 min, η^2^ = 0.034, 95% CI [0.000, 0.182]; difference = 179.78 W. Effect size and difference comparisons (see Tables 2, 3) indicated that the 4-min interval following the 90% 1RM chained deep squat intervention had the most pronounced impact on PP, with the greatest increase observed (η^2^ = 0.063,95% 95%CI [0.000, 0.228]; difference = 230.49 W).

### Peak rate of force development

The Shapiro-Wilk test confirmed that the data were normally distributed. A two-factor repeated measures ANOVA was conducted, and Mauchly’s test revealed a violation of the sphericity assumption (*P* < 0.01), prompting the use of multivariate test results. The analysis showed a significant effect of interval times on PRFD (F = 8.008 (5,64), *P* < 0.05, η^2^ = 0.385), but no significant interaction between interventions and interval times (F = 0.818 (15,198), *P* > 0.05, η^2^ = 0.048). Additionally, there were no statistically significant differences in CMJ PRFD between the two interventions (F = 0.291, *P* > 0.05, η^2^ = 0.013).

Pairwise comparisons (see Tables 2, 3; Figure 3C) revealed no significant differences in PRFD between pre-test and post-test values at any interval following either intervention (*P* > 0.05). According to effect size indicators and differences (see Tables 2, 3), the 4-min interval following the 90% 1RM traditional deep squat intervention had the most pronounced impact on CMJ PRFD, with the greatest increase observed (η^2^ = 0.034, 95% CI [0.000, 0.182]; difference = 679.94N/s).

### Peak impulses

The Shapiro-Wilk normality tests confirmed that the data followed a normal distribution, enabling the use of a two-factor repeated measures ANOVA. Mauchly’s test indicated a violation of the sphericity assumption (*P* < 0.05), leading to the prioritization of multivariate test results. A significant effect of interval times on CMJ PI was found (F = 14.69 (5,64), *P* < 0.05, η^2^ = 0.534). No significant interaction between interventions and interval times was detected (F = 0.946 (15,198), *P* > 0.05, η^2^ = 0.067), and no significant difference in PI was observed between the two interventions (F = 0.134, *P* > 0.05, η^2^ = 0.006).

The pairwise comparisons using the Sidak method (see Tables 2, 3; Figure 3D) revealed significant differences between pre-test and post-test values at the 4-min interval following the 90% 1RM CS intervention (*P* < 0.05; η^2^ = 0.019,95% CI [0.000, 0.150]; difference = 6.56N·s). Additionally, significant differences were observed at the 4-min and 8-min intervals following the 90% 1RM TS intervention (*P* < 0.05). For the 90% TS intervention, at 4 min, η^2^ = 0.010, 95% CI [0.000, 0.126]; difference = 4.87 N·s; at 8 min, η^2^ = 0.014, 95% CI [0.000, 0.138]; difference = 5.69 N·s. Effect size and difference comparisons (Tables 2, 3) indicated that the 4-min interval following the 90% 1RM CS intervention had the most pronounced impact on PI (η^2^ = 0.019, 95% CI [0.000, 0.150]; difference = 6.56N·s).

## Discussion

This study examined the acute effects of CS and TS on PAPE under a 90% 1RM load, assessing key performance indicators such as Jump Height (JH), Peak Power (PP), Peak Rate of Force Development (PRFD), and Peak Impulse (PI) during Countermovement Jump (CMJ). The results showed that both interventions effectively induced PAPE effects under high-intensity loading (90% 1RM), with CS performing better across multiple indicators, particularly exhibiting significant advantages within 4 min post-intervention.

Regarding Jump Height, both CS and TS showed significant improvements at 4 and 8 min post-intervention. The CS group exhibited a greater increase in JH (difference = 3.69 cm, η^2^ = 0.119), outperforming the TS group (difference = 2.44 cm, η^2^ = 0.059). This result is consistent with previous studies suggesting that high-intensity squats can enhance subsequent jump performance (Fukutani et al., 2014; Conrado de Freitas et al., 2021). It is noteworthy that the peak performance times differed between the two groups: the CS group reached its peak jump performance at 4 min post-intervention, whereas the TS group peaked at 8 min. This observation aligns with the optimal PAPE time window of 4.5–6.3 min reported in a recent meta-analysis (Xu et al., 2025), but also suggests that different squat modalities may influence the temporal characteristics of the PAPE response. Therefore, when designing pre-competition warm-up protocols or training plans, practitioners should consider the load characteristics and movement patterns of each exercise to appropriately determine recovery duration and maximize the potentiation effect. In terms of peak power (PP), both CS and TS interventions resulted in significant improvements. However, the CS group maintained elevated power levels from 4 to 16 min post-intervention and reached the maximum value at 4 min (difference = 230.49 W, η^2^ = 0.063). This finding indicates that CS not only induces PAPE effects earlier but also sustains them for a longer duration. Previous studies have demonstrated that variable resistance training effectively shortens the excitation–contraction coupling process, thereby allowing the muscle to produce maximal force more rapidly (Smith et al., 2019). According to the power calculation formula, producing high force output in a short period is critical for power enhancement (Owen et al., 2014). Therefore, CS may significantly improve explosive strength by enhancing force application efficiency within a short time frame. Nevertheless, despite the significant increase in PP observed at 12–16 min post-CS intervention, jump height (JH) did not show a corresponding improvement. This suggests that in the later recovery phase, although muscle power output remains elevated, its transfer efficiency to functional performance may decline. Thus, improvements in power output do not necessarily translate into enhanced jump height, which is ultimately governed by multiple factors such as take-off technique, force application patterns, and neuromuscular coordination (Gabriel et al., 2006).

Regarding peak impulse (PI), a significant increase was observed at 4 min post-CS intervention (difference = 6.56 N·s, η^2^ = 0.019), consistent with the improvements in JH and PP. TS also induced significant increases in PI at 4 and 8 min; however, the effect sizes were considerably smaller (difference = 4.87 N·s at 4 min, η^2^ = 0.010), further supporting the superiority of CS in enhancing propulsive force during take-off (Li et al., 2024). Notably, at 8 min post-CS intervention, despite a significant increase in JH, PI did not exhibit a statistically significant change. This suggests that PI alone may not fully explain the improvements in jump height, which also depends on more complex neuromuscular control mechanisms and the integration of force production (Gabriel et al., 2006).

In terms of peak rate of force development (PRFD), no significant changes were observed under either CS or TS conditions. This outcome diverges slightly from the findings of McLellan et al., who reported a strong positive correlation between PRFD and vertical jump performance (r = 0.68, *P* < 0.05) (Esformes et al., 2010), but aligns with Joseph et al. who found no significant changes in RFD following squat-based PAPE interventions (Esformes et al., 2010; Tseng et al., 2021). One plausible explanation is that in this study, PRFD was indirectly calculated using force–time curves from CMJ data, which have been shown to exhibit lower test-retest reliability (coefficient of variation: 7.9%–11.8%) compared to direct isokinetic assessment. Additionally, individual differences in strength level and training background may have contributed to the variability in RFD outcomes (Blazevich and Babault, 2019).

The results of this study demonstrate that both CS and TS can induce PAPE effects within 4–8 min post-intervention, thereby enhancing lower-limb explosive performance. These findings are consistent with previous research (Xu et al., 2025). Notably, CS elicited a more pronounced increase in jump height and impulse output at 4 min post-activation compared to TS, and its effect on power output was sustained for a longer duration. Given the critical role of explosive strength and acceleration in the initial 0–20 m phase of sprinting (Seitz and Haff, 2016), the peak performance index observed at 4 min post-CS intervention in this study (6.56 N·s) suggests that CS may better meet the biomechanical demands of sprint starts (Jiang and Xu, 2022). Therefore, it is recommended that sprinters perform 1–3 repetitions of CS at 90% 1RM prior to competition to optimize neuromuscular activation and improve sprint-specific explosive output. Additionally, CS may serve as a practical PAPE strategy within routine training cycles to enhance neuromuscular recruitment and initial acceleration capacity in sprint athletes.

However, through temporal analysis of performance metrics across various time points, we observed a gradual decline in the magnitude of PAPE effects induced by CS as recovery time increased. A similar trend was reported by Marín et al., who found that while vertical jump performance improved significantly at 2 min post-intervention, the potentiation effects progressively diminished over time (Marin et al., 2021). It is worth noting that the time points assessed in their study were earlier than those used in the current investigation. Supporting evidence also indicates that variable resistance training (VRT) can elicit significant PAPE responses within as little as 2 min or even earlier. For instance, Andrews et al. used a progressive VRT protocol involving Bulgarian split squats at 50%, 70%, and 90% 1RM, and reported significant improvements in CMJ performance at 1, 5, and 10 min post-activation (Andrews et al., 2016). Similarly, Fukutani et al.found that medium- and high-intensity squats induced significant CMJ height increases as early as 1 min post-exercise (Fukutani et al., 2014). These findings suggest that the time intervals chosen for post-activation testing may influence the observed PAPE trends, and may explain the gradual attenuation seen in the present study. Furthermore, most existing studies on VRT-induced PAPE have utilized either progressive-load traditional squats or band-assisted squat variations, which may introduce methodological variability. Therefore, future research should further explore the temporal pattern and underlying physiological mechanisms of CS-induced PAPE under short recovery intervals. Such investigations would provide more precise theoretical support for timing strategies and activation protocols in training and competition contexts.

## Limitations

Although previous studies have demonstrated that CS can effectively induce short-term PAPE, the specific mechanisms by which CS elicits PAPE under varying loading intensities remain insufficiently understood. The present study investigated the effects of CS only at a load of 90% 1RM, without examining the influence of different chain load ratios or a broader range of intensity conditions. Moreover, the study adopted a single fixed post-intervention recovery interval of 4 min, which may have limited the ability to accurately capture the optimal time window for peak performance enhancement. Given that previous literature has reported significant PAPE effects emerging at earlier time points (e.g., 2 or 3 min), future research should incorporate more frequent and closely spaced time intervals to dynamically monitor performance responses. This would allow for a more comprehensive evaluation of the time course of explosive performance enhancement following CS interventions.

Furthermore, current research on CS has primarily focused on acute, short-term effects, while comprehensive evaluations of its long-term training adaptations are still lacking. To better elucidate the mechanisms of CS-induced PAPE, future studies should investigate the neuromuscular regulation and physiological responses associated with CS across a range of intensity levels. Incorporating biochemical and physiological indicators—such as blood sample collection at multiple time points—may help clarify the temporal characteristics and underlying mechanisms of performance enhancement, thereby providing stronger theoretical support for evidence-based training strategies.

## Conclusions

Compared to TS performed at 90% 1RM, CS demonstrated a significant advantage in eliciting PAPE, with notable improvements in CMJ performance observed between 4 and 8 min post-intervention. Overall, CS elicited a stronger PAPE response under high-intensity loading conditions. A key benefit of CS lies in its ability to apply progressively increasing external resistance during the ascent phase, which helps to mitigate the mechanical limitations associated with the “sticking point” and optimize force output.

The findings of this study suggest that CS is not only an effective form of conditioning contraction (CC) capable of enhancing neuromuscular adaptation to high-intensity loads, but also a practical pre-competition warm-up strategy for improving lower-limb explosive power. This approach holds clear practical value and provides a feasible theoretical basis for individualized, task-oriented training and warm-up program design in competitive sports.

## Data Availability

The raw data supporting the conclusions of this study are included in the article or supplementary material. For further inquiries, please contact the corresponding author.
